# Sclerotial Formation of *Polyporus umbellatus* by Low Temperature Treatment under Artificial Conditions

**DOI:** 10.1371/journal.pone.0056190

**Published:** 2013-02-20

**Authors:** Yong-Mei Xing, Li-Chun Zhang, Han-Qiao Liang, Jing Lv, Chao Song, Shun-Xing Guo, Chun-Lan Wang, Tae-Soo Lee, Min-Woong Lee

**Affiliations:** 1 Institute of Medicinal Plant Development, Chinese Academy of Medical Sciences & Peking Union Medical College, Beijing, People’s Republic of China; 2 Department of Life Science, University of Incheon, Incheon, Korea; 3 Department of Life Science, Dongguk University, Seoul, Korea; College of Tropical Agriculture and Human Resources, University of Hawaii, United States of America

## Abstract

**Background:**

*Polyporus umbellatus* sclerotia have been used as a diuretic agent in China for over two thousand years. A shortage of the natural *P. umbellatus* has prompted researchers to induce sclerotial formation in the laboratory.

**Methodology/Principal Finding:**

*P. umbellatus* cultivation in a sawdust-based substrate was investigated to evaluate the effect of low temperature conditions on sclerotial formation. A phenol-sulfuric acid method was employed to determine the polysaccharide content of wild *P. umbellatus* sclerotia and mycelia and sclerotia grown in low-temperature treatments. In addition, reactive oxygen species (ROS) content, expressed as the fluorescence intensity of mycelia during sclerotial differentiation was determined. Analysis of ROS generation and sclerotial formation in mycelia after treatment with the antioxidants such as diphenyleneiodonium chloride (DPI), apocynin (Apo), or vitamin C were studied. Furthermore, macroscopic and microscopic characteristics of sclerotial differentiation were observed. Sclerotia were not induced by continuous cultivation at 25°C. The polysaccharide content of the artificial sclerotia is 78% of that of wild sclerotia. In the low-temperature treatment group, the fluorescent intensity of ROS was higher than that of the room temperature (25°C) group which did not induce sclerotial formation all through the cultivation. The antioxidants DPI and Apo reduced ROS levels and did not induce sclerotial formation. Although the concentration-dependent effects of vitamin C (5–15 mg mL^−1^) also reduced ROS generation and inhibited sclerotial formation, using a low concentration of vitamin C (1 mg mL^−1^) successfully induced sclerotial differentiation and increased ROS production.

**Conclusions/Significance:**

Exposure to low temperatures induced *P. umbellatus* sclerotial morphogenesis during cultivation. Low temperature treatment enhanced ROS in mycelia, which may be important in triggering sclerotial differentiation in *P. umbellatus*. Moreover, the application of antioxidants impaired ROS generation and inhibited sclerotial formation. Our findings may help to provide new insights into the biological mechanisms underlying sclerotial morphogenesis in *P. umbellatus*.

## Introduction

*Polyporus umbellatus* (Pers.) Fr., one of the most precious and widely used medicinal fungi, belong to the Polyporaceae family of Basidiomycota phylum [1]–[2]. *P. umbellatus* sclerotia have been shown to possess pharmacological activities for the treatment of conditions such as acute nephritis and edema [3]. Additionally, the antitumor properties of polysaccharides isolated from *P. umbellatus* sclerotia have been well documented for over 30 years [4]. Recently, many reports have focused on the treatment of certain cancers, including leukemia [5], liver cancer [6], using active constituents isolated from *P. umbellatus*. Furthermore, treatment with polysaccharides from *P. umbellatus* was shown to effectively alleviate patients’ symptoms and inhibit DNA reproduction of the pathogenic virus in curing hepatitis B [7]–[8]. *Polyporus umbellatus* polysaccharide has also been demonstrated to possess the immunostimulating, anti-inflammatory and hepatoprotective properties [9]–[13].

However, wild sclerotia of *P. umbellatus* have been largely depleted due to insufficient protection, over-harvesting and severe habitat loss [3]. Therefore, interest in the mass production of *P. umbellatus* under artificial conditions has increased in recent years. Although semi-artificial cultivation of *P. umbellatus* via infection with *Armillaria mellea* has been practiced over the past 30 years, this technique is restricted by low proliferation rate, unstable yield and the lack of natural sclerotia to serve as seeds [14]. This situation has given rise to an interest in producing sclerotia of *P. umbellatus* directly from hyphae instead of from sclerotia in the laboratory settings.

In the previous studies, sclerotial formation was successfully induced by culturing *P. umbellatus* in Petri dishes containing fructose [14], maltose and glucose complete medium [3]. The carbon source and initial pH values were considered to be essential factors for sclerotial formation in *P. umbellatus*. In nature, *P. umbellatus* sclerotia grow underground and create symbiotic relationship with *A. mellea* near the root of the birch, oak or the maple tree in the mountains [15]. Thus, the fungal species growing in nutrient-supplemented sawdust substrates was more similar to the natural conditions than that growing in Petri plates containing nutrient agar. Sclerotia produced in nutritional agar medium might be less of practical significance than that generated in sawdust-based medium. Therefore, in this study, we investigated sawdust-based cultivation of *P. umbellatus* sclerotia under low temperature conditions.

Sclerotia are presumed to form from a hardened mass of mycelia when the organism is subjected to harsh environmental conditions of dryness, cold, drought or nutritional starvation or other conditions that are hostile to growth [16]. In *Sclerotium rolfsii*, physical, chemical or nutritional factors have been reported to affect sclerotial formation [17]. In 1991, Carroll confirmed that short-term exposure to low temperatures induced sclerotial development in *S. rolfsii* [18]. In the study, a *S. rolfsii* Sacc. isolate was cultured using two different media glucose/yeast extract and glucose/ammonium nitrate in Petri dishes at 24°C, and the resulting colonies were subjected to a short cold treatment (3 hours at 5°C). Sclerotia appeared in a ring where the colony margin was exposed to cold shock.

Reactive oxygen species (ROS) are chemically reactive molecules that are normal products of cellular metabolism. ROS include hydroxyl radicals, alkoxyl, alkoperoxyl radicals and singlet oxygen, etc in biological systems. ROS are known to play important roles in homeostasis and cell signaling [19]–[20]. ROS levels are commonly regulated by antioxidant mechanisms that consist of enzymatic and nonenzymatic systems. However, in cases of environmental stress, ROS levels can increase dramatically. Harmful levels of ROS, known as oxidative stress states, can be caused by imbalances in antioxidant defenses [21] and can result in considerable damage to organisms.

In *S. rolfsii*, Georgiou was the first to demonstrate that sclerotial differentiation was closely associated with a high degree of lipid peroxidation [22]. In other words, sclerotial differentiation is triggered by oxidative stress. Later, it was shown that sclerotial biogenesis in *S. rolfsii*, *Sclerotinia minor* and *Rhizoctonia solani* could be inhibited by reducing oxidative stress [23]–[24]. Recently, sclerotial differentiation in *S. minor* was demonstrated to depend on thiol redox state and oxidative stress [25]. Moreover, β-carotene inhibits sclerotial differentiation [26], and ascorbic acid (vitamin C) delays sclerotial differentiation in a concentration-dependent manner [27]. Subsequently, researchers have begun to focus on the mechanisms underlying sclerotial differentiation in phytopathogenic fungi. However, very few reports are available on sclerotial differentiation in medicinal Basidiomycota phylum.

The aim of the present study was to examine the effects of low temperatures on sclerotial formation in sawdust-based media and explore the relationship between ROS and sclerotial differentiation in *P. umbellatus*. The results from this study will help to further our understanding of the mechanisms underlying low temperature-induced sclerotial transformation. Additionally, these results will establish a basis for further investigation into the relationship between oxidative stress and sclerotial formation in *P. umbellatus*.

## Results

### Different Stages of Sclerotial Formation are Induced by Low Temperature Cultivation

**Figure 1:**
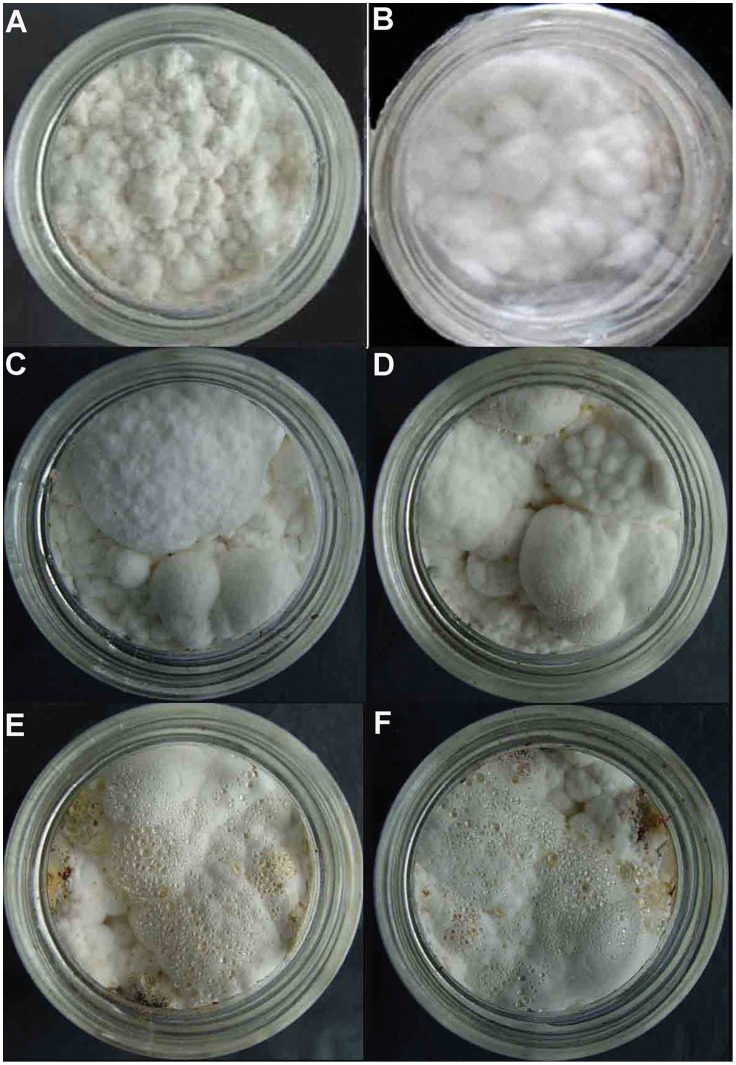
Different stages of sclerotia induced by low-temperature vs. mycelia in *P. umbellatus* cultivated at 25°C. (A) Mycelia could not form sclerotia after cultivation at 25°C for 105 days in the control group. (B) SI stage of sclerotial differentiation at 60 days after cultivation in the low-temperature group. (C) and (D) SD stage of sclerotial formation at 90 days after cultivation in the low-temperature group. (E) and (F) SM stages of sclerotial formation with the total cultivating of 105 days in the low-temperature group. Representative images were from three independent experiments, with 30 replicates in each group.

In the control group, *P. umbellatus* did not form sclerotia when cultivated at 25°C during 105 days (Figure 1A). However, in the low temperature group, sclerotial initiation in the form of snow-white interwoven hyphae with abundant aerial mycelia appeared at day 60 of cultivation; at this time point, the fungi had been cultivated at 5°C for 15 days. This stage was termed the sclerotial initiation (SI) stage (Figure 1B). On the 90^th^ day, the initial sclerotia with spherical or irregular forms increased in size and were often accompanied with transparent exudate droplet; furthermore, the surface color turned to grey-white, and the mycelia on the surface were much denser compared with the SI stage. This stage of development was termed the sclerotial development (SD) stage (Figure 1C and D). In the sclerotial maturation (SM) stage, brown pigment was unevenly deposited on the surfaces of the interwoven hyphae and was always accompanied by brown exudate droplet (Figure 1E and F). Thus, three successive stages of sclerotial formation were observed in the low temperature group.

However, if we cultured *P. umbellatus* exclusively at 5°C for 105 days from the beginning to the end, the mycelia grew very slowly and no sclerotia formed. After 45 days’ cultivation at 25°C, if we transferred *P. umbellatus* to 5°C and cultivated the fungus continuously at this temperature for 60 more days, small sclerotia formed on 105^th^ day (data not shown). In addition, only the SI stage of sclerotia formation was observed in the latter case.

### Polysaccharide Content in Wild *P. umbellatus* Sclerotia and *P. umbellatus* Mycelia and Sclerotia Grown under Artificial Conditions

The polysaccharide content of the wild sclerotia reached as high as 6.33%, which was 1.62 times that of the mycelia grown under low temperature conditions and 1.28 times that of the sclerotia induced by low temperature cultivation (P<0.05). In addition, the total carbohydrate content of the artificial sclerotia was 1.27 times that of the hyphae induced by low temperature treatment (P<0.05) (Table 1). We also found that the polysaccharide content of the sclerotia cultivated in sawdust-based substrate was much higher compared to the sclerotia cultivated in fructose, maltose and glucose complete agar medium in a previous study [3] (data not shown).

**Table 1:** Polysaccharide content in wild sclerotia, artificial sclerotia and mycelia of *P. umbellatus*.

Group (n = 10)	Polysaccharide content
Wild sclerotia	6.33%±0.09%*
Sclerotia induced by low-temperature of 5°C	4.94%±0.12%*
Mycelia induced by low-temperature of 5°C (control)	3.90%±0.10%

The standard curve equation of D-glucose was y = 10.31x+0.0052, R^2^ = 0.9994, which indicated that within a D-glucose concentration range from 0 to 160 µg/ml, the corresponding absorbance values exhibited a reliable linear relationship. Data were analyzed with one-way ANOVA. Significant differences were determined using the Student-Newman-Keuls method. The values were presented as the means ± SD from at least three independent experiments, with 10 replicates of each group, *P<0.05 (compared to the control group).

### ROS Analysis in *P. umbellatus* Mycelial Cells

**Figure 2:**
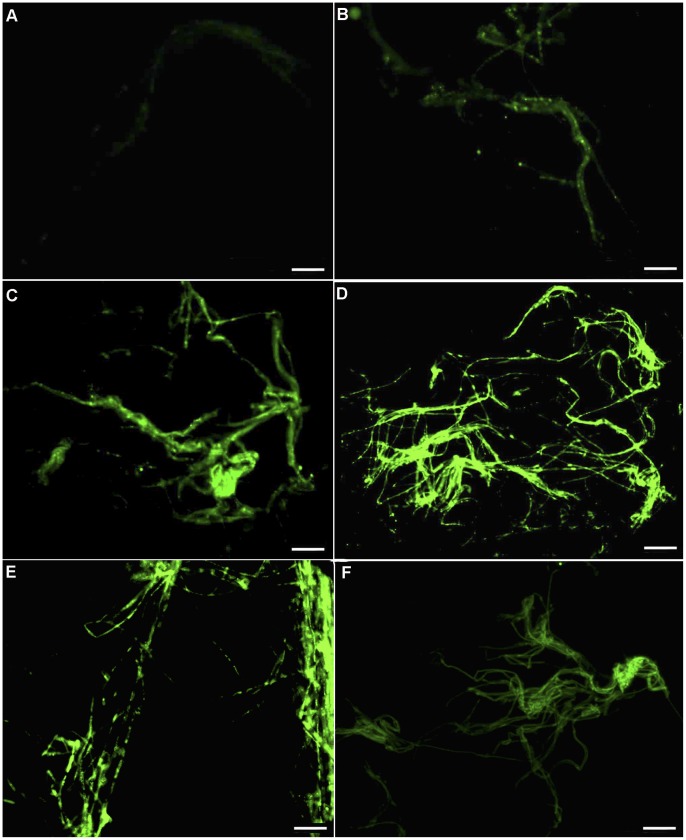
Fluorescent image of ROS in *P. umbellatus* mycelial cells in the low temperature group. ROS accumulation measured by CM-H_2_DCFDA staining of mycelia was observed at different time periods of cultivation. (A), (B), (C), (D), (E) and (F) respectively represented ROS content in mycelia after 45, 60, 75, 90, 90 and 105 days after cultivation in low-temperature group. These pictures were captured using a Zeiss fluorescent microscope at the 400×magnification. Scale bar, 5 µm. Representative images were from three independent experiments, with 50 replicates during each cultivation stage.

In the low temperature group, the intracellular ROS levels were low after 45 days of cultivation at 25°C (Figure 2A); however, the ROS levels increased after 15 days of cultivation at 5°C, which corresponded with the SI stage (Figure 2B). When the cultures were transferred back to 25°C for cultivation, mycelial intracellular ROS levels rose sharply on the 75^th^ day (Figure 2C). On the 90^th^ day, which corresponded to the SD stage of sclerotial formation, the ROS content in mycelial cells reached a maximum level (Figure 2D and E); ROS levels then decreased slightly during the SM stage (day 105) (Figure 2F).

**Figure 3:**
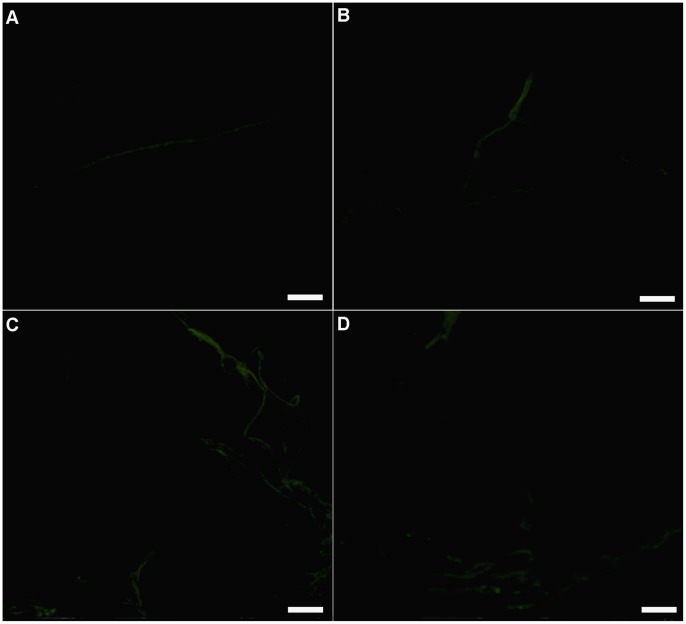
Fluorescent images of ROS in the mycelial cells of *P. umbellatus* at 25°C. Mycelial ROS content was observed at different cultivation stages using CM-H_2_DCFDA staining. (A), (B), (C) and (D) respectively represented ROS levels in mycelia after 45, 75, 90 and 105 days of cultivation in the room temperature (control) group. These pictures were also taken under a Zeiss fluorescent microscope at the 400×magnification. Scale bar, 5 µm. Representative images were from three independent experiments, with 50 replicates during each cultivation stage.

In the room temperature group, which did not form sclerotia, ROS levels in the mycelial cells remained low throughout the 105 day cultivation period (Figure 3).

**Figure 4:**
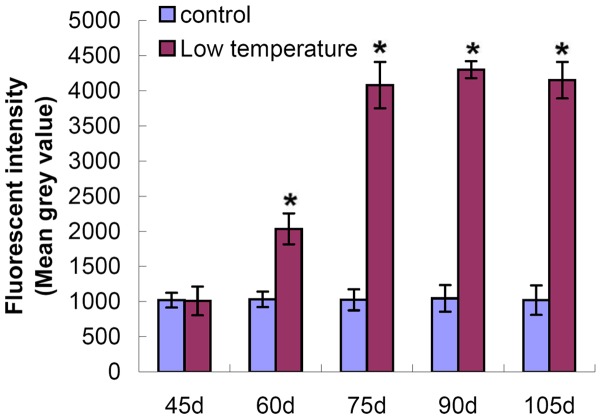
ROS analysis of mycelial cells in the room temperature group vs. low temperature group. The room temperature group was used as the control group. Independent T-test was used to analyze the data of the two groups at each cultivation phase. The data presented represent means ± SD from three independent experiments, with 50 replicates in each group.*P<0.01.

It was showed that ROS content increased sharply after low temperature treatment, whereas ROS levels in the fungus maintained at 25°C remained low throughout the entire cultivation period (Figure 4).

### Analysis of ROS Production and Sclerotial Formation in Mycelia after Treatment with Different Antioxidants

After cultivation for 120 days, no sclerotia formed in the groups treated with DPI, Apo and high concentration of vitamin C (≥5 mg mL^−1^). Mycelia treated with a low concentration of vitamin C (1 mg mL^−1^) formed sclerotia; however, the biomass of the sclerotia is only 60% of that observed in the low-temperature group without antioxidants treatment (data not shown).

**Figure 5:**
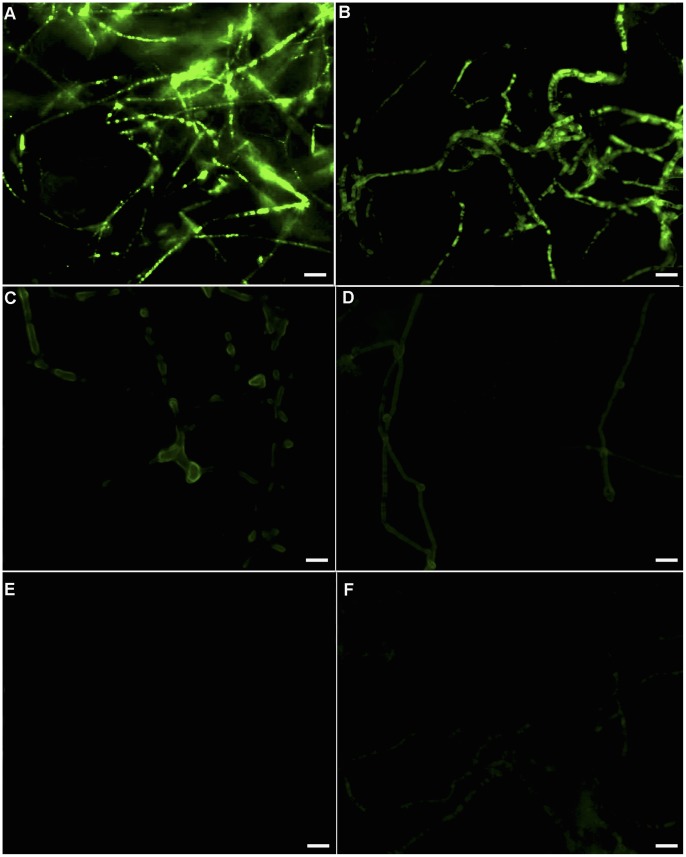
ROS images in low temperature group vs. different antioxidants groups after 90 days of cultivation. (A) Fluorescent image of ROS in low temperature group (control). (B) VitaminC (1 mg mL^−1^ ) complemented group. (C) Group treated with vitaminC (5 mg mL^−1^ ). (D) Group complemented with vitaminC (10 mg mL^−1^ ). (E) VitaminC (15 mg mL^−1^ ) complemented group. (F) Fluorescent image of ROS in DPI (1 mM) or Apo (10 mM ) complemented group. The pictures were taken using a Zeiss fluorescent microscope. Images were representatives of three independent experiments, with 30 replicates in each group. Scale bar, 10 µm.

The sclerotial mycelia generated the largest amounts of ROS in the low-temperature group after cultivation for 90 days (Figure 5A), while the mycelia in the groups treated with antioxidants, such as vitamin C (5, 10 and 15 mg mL^−1^) (Figure 5C, D and E), DPI or Apo (Figure 5F) impaired sclerotial development in *P. umbellatus* and neutralized the effects of ROS. Vitamin C (5, 10 and 15 mg mL^−1^) affected ROS generation in a concentration-dependent manner. The reduced ROS content was accompanied by an increased vitamin C concentration. When complemented with vitamin C (15 mg mL^−1^), the ROS fluorescent intensity was hardly detectable (Figure 5E). However, when complemented with a low concentration of vitamin C (1 mg mL^−1^), sclerotial formation was induced, but the ROS fluorescent intensity (Figure 5B) is lower than that observed for sclerotial formation under low temperatures (Figure 5A), and higher than that observed for the antioxidant complemented groups that did not produce sclerotia (Figure 5C, D, E and F).

### Microscopic Observation of *P. umbellatus* Mycelia during Sclerotial Formation under Low Temperatures

**Figure 6:**
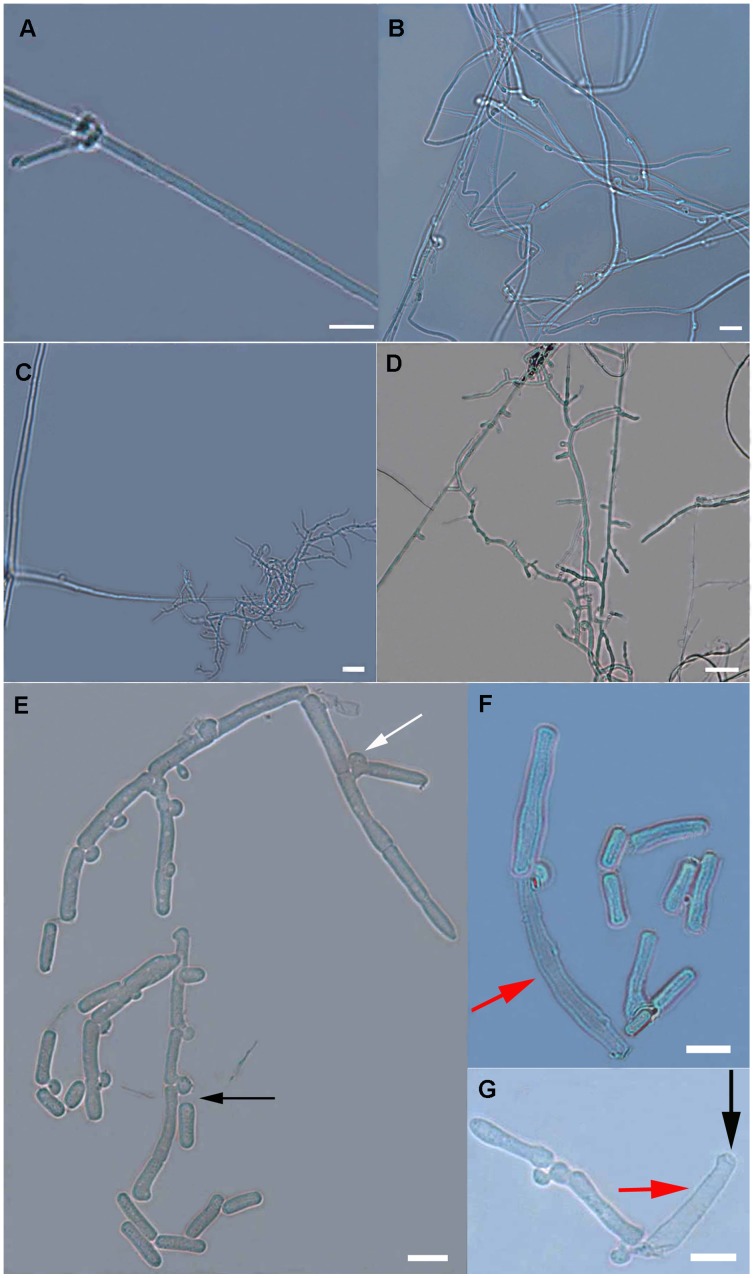
Microscopic characteristics of *P. umbellatus* mycelia during sclerotial formation. *P. umbellatus* mycelia was subjected to morphological examination using Zeiss Axio Imager A1 microscope. (A) Mycelia with little branches at 30 days of cultivation. (B) Mycelia with multiple clamp connections 60 days after inoculation. (C) Thick and slim mycelia coexited at 90 days after inoculation. (D) Slim mycelia with multiple branches after 120 days of cultivation. (E) Colorless conidia and spore-producing structures were observed at 120 days of cultivation. (F) and (G) indicated the sclerine of the conidia (red arrows). Representative images were from three independent experiments. Scale bar, 10 µm.

Mycelia with small branches were observed after 30 days of inoculation (Figure 6A). At 60 days, the mycelia grew vigorously with multiple clamp connections (Figure 6B). Thick mycelia and slim mycelia with multiple branches were observed after 90 days of cultivation (Figure 6C). The diameters of the thick hyphae ranged from 1.52 µm to 2.18 µm, while the slim hyphae were only from 0.98 µm to 1.13 µm in diameter. At 120 days after inoculation, slim mycelia with multiple branches were predominately observed (Figure 6D). In addition, the formation of colorless conidia (arthrospores) was observed concomitantly with the appearance of slim mycelia (Figure 6E). Spore-producing structures (Figure 6E) and the conidia, which were individually fractured from the mycelial clamp connections, were also observed. The clamp connections and the arthrospores were dissociated (Figure 6E black arrow). The conidia-forming mycelial branches were held together through the clamp connections (Figure 6E white arrow). The size of the conidia ranged from 9.01 µm to 20.05 µm in length, with a diameter of approximately 5.00 µm. In addition, the *P. umbellatus* arthrospores were considered as enteroarthric, but not holoarthric conidium, and the conidia and the outer sclerine (Figure 6F and G red arrow) were separated. These results indicated that during arthrospore formation, the mycelia generated transverse septums, and the mycelial sclerine did not participate in conidial development. Moreover, we observed a hole at one end of the scerine (Figure 6G black arrow) and the dissociation of arthrospores from the other end of the sclerine.

### Scanning Electron Microscopic Examination of the Artificial Sclerotia after Cultivation for 120 Days

**Figure 7:**
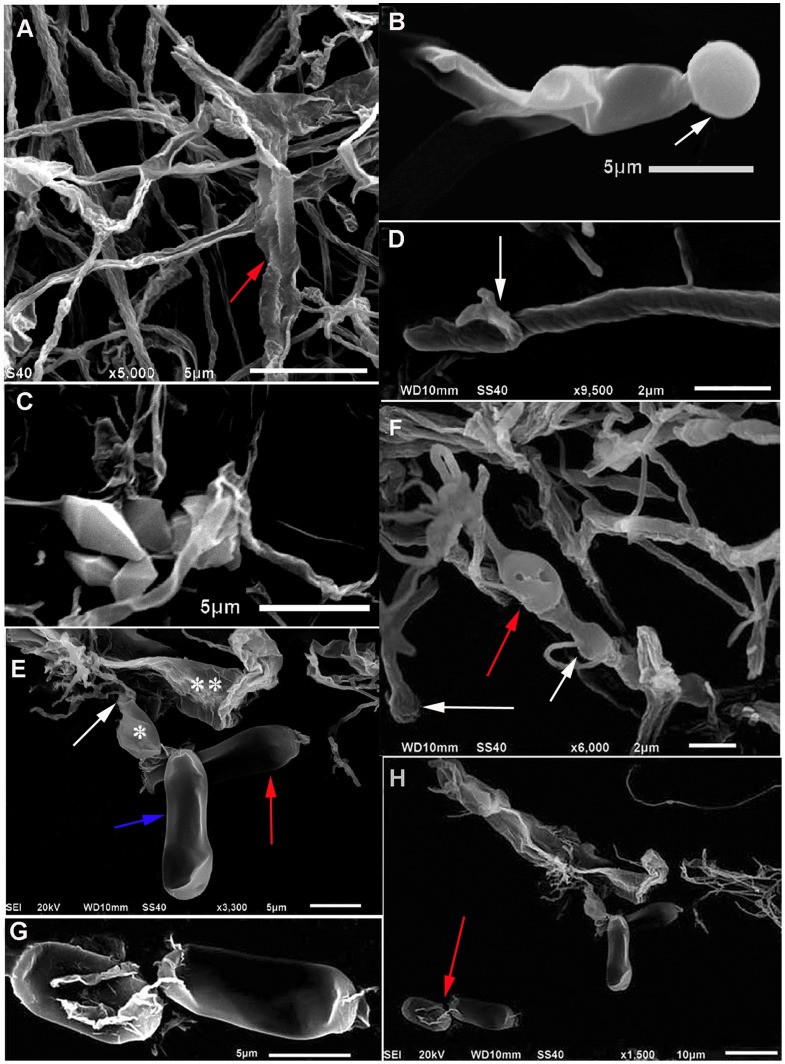
Observation of *P. umbellatus* sclerotia by SEM 120 days after cultivation. The pictures were observed using a JEOL JSM-6510 Scanning Electron Microscope. (A) Hyphae intertwined and fused were observed (red arrow). (B) Inflated mycelia like a ball at one end. (C) A cluster of multilateral crystalline structures. (D) Helical strikes on the surface of the mycelium with clamp connections (white arrow). (E) Conidia (arrows) and sclerine (* and **). (F) Local parts of mycelia swollen (arrows). (G) Sclerine residue on the surface of additional conidia. (H) Conidia formed with surrounding slim mycelia. Images were representatives of three independent experiments. Scale bar, (A), (B), (C), (E) and (G) 5 µm; (D) and (F) 2 µm; (H) 10 µm.

Scanning electron microscopy (SEM) was used to further examine the arthrospores (Figure 7E, G and H). Hyphal branching and hyphae fusion were observed at 120 days after inoculation (Figure 7A). The hyphae were intertwined and fused like ropes (Figure 7A red arrow), and the swollen hyphae appeared as balls at the end of the mycelia (Figure7B white arrow). Aggregated multilateral structures were observed suggesting the formation of calcium oxalate crystals (Figure 7C). Helical stripes covered on the mycelial surface (Figure 7D) with clamp connection (Figure 7D white arrow). The mycelia were swollen in local areas (Figure 7F arrows), with pits on the surface of the deflated structures (Figure 7F red arrow). The conidiospores were surrounded with slender and multilevel-branching mycelia (Figure 7H). The conidia were cylindrical or barrel-shaped, with one obtuse side and one subacute or irregularly shaped side. The lower conidium (Figure 7E red arrow) was dissociated from the adjacent residual outer sclerine (Figure 7E*). The lower conidium (Figure 7E red arrow) and the outer sclerine (Figure 7E*) were similar in appearance. A hole was observed at the upper end of the outer sclerine (Figure 7E white arrow), consistent with the previously described microscopic observations (Figure 6G black arrow). An additional outer sclerine (Figure 7E**) was observed above the two conidia. We also observed that residual material from the outer sclerine appeared on the surface of two additional conidia (Figure 7G and H red arrow).

### Microscopic Structure of *P. umbellatus* Sclerotia Using Paraffin Sectioning

**Figure 8:**
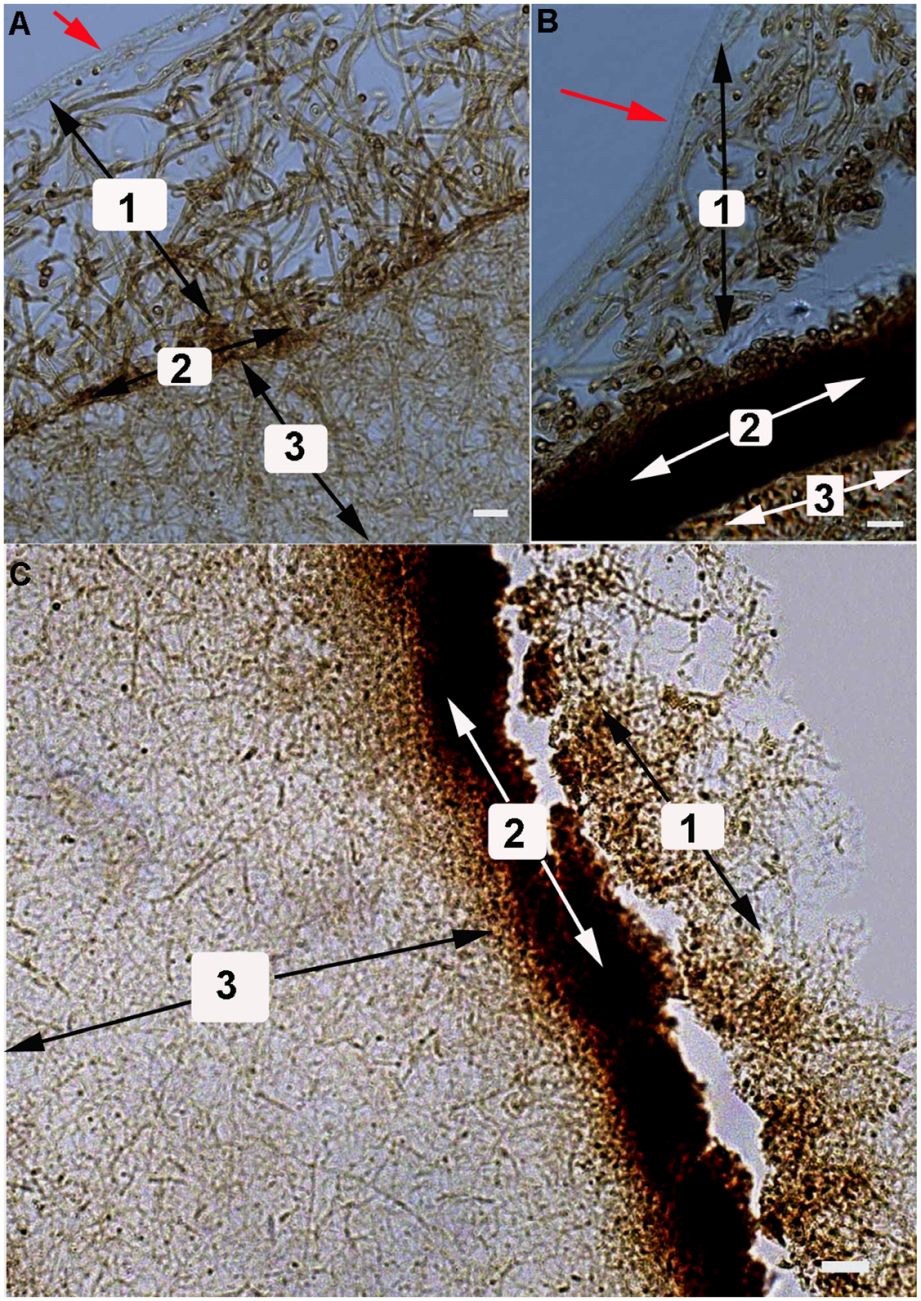
Microscopic structure of *P. umbellatus* artificial sclerotia at different stages of cultivation. The pictures were examined using Zeiss Axio Imager A1 microscope. (A) The cultivation of artificial sclerotia for 105 day. (B) The cultivation of artificial sclerotia for 120 days. The outermost membrane in (A) and (B) was indicated with a red arrow. (C) The cultivation of artificial sclerotia for 150 days. 1 represented the loose hyphae near the outermost membrane, 2 represented the pigmented layer, and 3 represented the thick and interwoven hyphae. Representative images were from three independent experiments. Scale bar, (A) and (B) 10 µm; (C) 20 µm.

Three layers of microscopic *P. umbellatus* sclerotial structures, indicated as 1, 2 and 3 in Figure 8, were observed using paraffin sectioning at different cultivation stages (at 105, 120 and 150 days). After cultivation for 105 days, the outermost structure appeared as a spindle membrane of layer (Figure 8A red arrow) which might protect the inner structures of the sclerotia. Loosely woven hyphae were observed in the inner area (Figure 8A–1) close to the spindle membrane. This area would continuously develop more hyphae eventually forming a volumetric growth of sclerotia. A thin and pigmented layer was observed near the loosely woven hyphae (Figure 8A–1), which would eventually develop into the hardened and blackish epidermal outer surface (Figure 8A–2), growing larger and thicker with subsequent passaging. The sclerotia also contained thick and interwovened hyphae that remain as a dense fungal tissue mass during later stages of growth (Figure 8A–3).

After cultivation for 120 days, we observed that the region of loosely woven hyphae (Figure 8B–1) and the outermost spindle membrane layer (Figure 8B red arrow) were being replaced with the pigmented part. The pigmented layer of epidermal structure grew thicker in volume (Figure 8B–2), and the thick and interwoven hyphae became denser (Figure 8B–3). After cultivation for 150 days, the outer layer transformed into aerial hyphae, and the outermost spindle membrane layer completely disappeared (Figure 8C–1). Thus, we have identified the microscopic structures of artificial sclerotial morphogenesis in *P. umbellatus*.

## Discussion

In this study, temperature shift treatment was used to induce the formation of *P. umbellatus* sclerotia. After growing *P. umbellatus* at 25°C for 45 days in sawdust medium, the fungus was cultivated at 5°C for 15 days and returned to 25°C to induce sclerotia formation. After screening, we concluded that these conditions were optimal for *P. umbellatus* sclerotial development. These results might provide new insight into the mass production of *P. umbellatus* sclerotial formation.

*P. umbellatus* sclerotial development at different temperatures under natural conditions shows similar metamorphic phenomenon as those observed in this study. Previously, *P. umbellatus* sclerotia were shown to go into dormancy underground below 8°C in the late autumn and to start germinating at temperatures above 8°C. Additionally, *P. umbellatus* sclerotia grow quickly at temperatures above 12°C in their natural habitat [28].

Based on our findings, we hypothesize that sclerotia cannot grow normally in the absence of natural temperature variation. In other words, temperature shifts might play an important role in natural *P. umbellatus* sclerotial biogenesis.

Sclerotia cultivated in a sawdust-based substrate produced much higher levels of polysaccharides than sclerotia grown in nutritional agar medium, indicating that the sawdust-based media will be of great use in further studies. Additionally, polysaccharide concentration of sclerotia grown under artificial conditions was greater compared with *P. umbellatus* mycelia (*P*<0.05) (Table 1). These results suggest that it could be useful to induce sclerotia from mycelia. The information from this study will help to establish a basis for mass production of *P. umbellatus* sclerotia.

Fungi, as a group of the eukaryotes, are inevitably subjected to different environmental stresses such as starvation, temperature variation and ionizing radiation throughout their lives in their natural habitats [29]. These stresses can induce oxidative stress in fungal cells, which may provoke morphological differentiation. *Neurospora crassa* was demonstrated to form microconidia, macroconidia or ascospores in response to external stress factors of starvation or light [30]. In addition, in eukaryotic microorganisms, cell differentiation is known to be associated with increased ROS levels [31].

In this study, exposure to low temperatures during cultivation induced *P. umbellatus* to enter into the state of oxidative stress. ROS concentrations of mycelial cells in *P. umbellatus* cultivated at 5°C were higher than in *P. umbellatus* cultivated at 25°C. This may be due to the fact that cold temperatures, which promote sclerotial formation, can increase oxygen solubility in the cytoplasm of fungi cultivated in growth medium or water [32]. However, if we cultured *P. umbellatus* continuously at 5°C after 45 days of cultivation at 25°C, small sclerotia formed during the SI stage (data not shown). A possible explanation for this phenomenon is that the mycelia and sclerotia grew very slowly under continuously cold conditions. On the other hand, as abundant mycelial growth is a prerequisite for sclerotial differentiation, the slow growth inhibited sclerotial development [14]. In addition, slow growth, growth ceases and morphogenetic changes are also common responses to oxidative stress [30], [33]. When *P. umbellatus* were returned to 25°C, sclerotia developed gradually. It is likely that the temperature shift further increased intracellular ROS in *P. umbellatus* mycelia. Once the ROS levels increased, the hyperoxidant state becomes more severe. As a result, ROS in *P. umbellatus* mycelial cells accumulated to such an extent that the fungus formed sclerotia to adapt to the new conditions by means of reducing intracellular oxygen concentrations and keeping away from the oxidative stress state. [33]. ROS are generated at the start and during the period of cell differentiation in certain eukaryotic organisms [31]. In the present study, ROS concentration started to rise after low temperature treatment and kept at high levels throughout the different stages of sclerotial formation (SI, SD and SM) (Fig. 2, Fig. 4).

The ROS content in *P. umbellatus* mycelial cells remained low in samples that were incubated at 25°C continuously, indicating that the low oxygen concentrations in the mycelial cells failed to trigger the generation of a hyperoxidant stress condition. Consequently, no sclerotia formed during cultivation in the control group. Our findings indicated that high oxidative stress induced the condensation and fusion of *P. umbellatus* mycelia in response to the adverse conditions. At the same time, a lack of antioxidant enzymes or sclerotia-inhibiting substances [34], which were known as hydroxyl radical scavengers [35] leads to higher ROS levels that promote cytodifferentiation [31]. As a result, hyphae of *P. umbellatus* aggregated, fused, branched and melanized step by step, which led to sclerotial formation.

Faced with hyperoxidative stress, organisms rely on antioxidant mechanisms to neutralize accumulating ROS. One method for microorganisms to prevent significant oxidation-induced damage is to generate antioxidant substances, such as vitamin E, oxalic acid, ascorbic acid and antioxidant enzymes such as superoxide dismutase [36], catalase, and peroxidase as well [32]. As the exudate that appeared on the surface of the sclerotia comprised oxalic acid [37]–[38] and antioxidant enzymes [31], these antioxidant molecules might play a part in resisting the hyperoxidant state. In the present study, exudate deposited on the surface of the *P. umbellatus* sclerotia in the SD and SM stage. In addition, the prevention of oxidative stress requires hyphal branching and fusion or cell aggregation to occur, which are the main processes involved in sclerotial formation [34], [39].

The antioxidant complement study revealed that a low-concentration of vitamin C (1 mg mL^−1^) did not fully compensate the fluorescent intensity of ROS, and the ROS content was higher than in groups treated with a high concentration of vitamin C, DPI or Apo. In addition, only 60% sclerotial biomass formed compared to the low-temperature group, suggesting that sclerotial formation is associated with high oxidative state. Consistent with these data, antioxidants such as DPI and *N*-acetyl-cysteine (NAC) impaired sclerotial development in *S. sclerotiorum*. Staining with 2′7′-dichlorodihydrofluorescein diacetate (DCHFDA) indicated that the fluorescence emission of ROS was higher in sclerotial initials of *S. sclerotiorum*. These results showed that sclerotial initiation was associated with the generation of intracellular ROS. This conclusion is consistent with the data that inhibitors of ROS, DPI or NAC considerably reduce sclerotial initiation and completely inhibit sclerotial maturation [40].

While it has been reported that ROS are essential for the developmental process during cell differentiation [31], our findings provide evidence for the first time that increased oxidative stress levels may be associated with sclerotial formation in a medicinal fungus belonging to the Basidiomycota phylum. Oxidative stress in relation to sclerotial formation has been reported in phytopathogenic ascomycetes [42] .

After cultivation for 90 days or 120 days, the thick mycelia were almost replaced with slim mycelia, and the appearance of arthrospores and sclerotia was observed. These results might reflect nutrition depletion, as both conidia and sclerotia formed at times of extreme stress. Hyphae fusion and branching were considered to be important processes in sclerotial formation [39].

In the present study, hexagonal or octahedral crystalline structures of artificial sclerotia in *P. umbellatus* were observed using SEM (Figure 7C), which was similar to the hexagonal structure that Guo and Xu observed using light microscopy [41]. Irregular crystalline structures were previously described in *S. sclerotium* using SEM [39]. The formation of localized crystalline structures and curled hyphae were one of the prominent characteristics in *S. sclerotium* sclerotial formation. The microscopic observation of sclerotial structures after cultivation for 150 days using paraffin sectioning indicated that artificial sclerotia had structures that were similar to those of natural sclerotia [41].

In terms of the morphological differentiation that occurs during sclerotial formation in *P. umbellatus*, the identification of the characteristics of sclerotial differentiation obtained here will facilitate further studies focusing on the basic physiological process of sclerotial formation.

The present study highlighted the relationship between conidia formation and clamp connections in arthrospores production structure during sclerotial formation in sawdust medium. Furthermore, the arthrospore was considered to be enteroarthric not holoarthric conidium for the first time. The results obtained from the morphological analysis of sclerotia and mycelia during cultivation will provide new insight into the mechanism underlying the growth of *P. umbellatus*.

The information obtained in this research might be of great use in considering the conservation and mass production of artificial sclerotial formation in *P. umbellatus*. Additional studies will be conducted to examine other active ingredients in sclerotia produced under artificial conditions. It may also prove valuable to explore the symbiotic relationship in nature between *A. mellea* and *P. umbellatus* sclerotia that are induced under artificial conditions.

## Materials and Methods

### Ethics Statement

1. All of the samples described in the manuscript were collected from Dangjiashan village, Beiping town, Guxian of Shangxi province, China.
2. An experimental base was designated by the Chinese government as the cultivation field of *P. umbellatus* which was in charge of Institute of Medicinal Plant Development, Chinese Academy of Medical Sciences & Peking Union Medical College in the mid 1980s. It was managed by Professor Jin-Tang Xu more than 20 years ago. After Xu retired, it was supervised by Professor Shun-Xing Guo, who was one of the co-authors in our manuscript.
3. At the same time, wild sclerotia of *P. umbellatus* was also collected for research from the experimental base mentioned above. Thus, no specific permits were required for the described field studies. The fungal species of *P. umbellatus* was not the protected species in China.
4. The field studies did not involve any endangered or protected species.

### Specimen

The *P. umbellatus* used in this study was isolated from wild triennial sclerotia collected from Dangjiashan village, Beiping town, Guxian of Shangxi province, China by Shun-Xing Guo. Cultures were maintained on wheat bran agar slants medium (wheat bran (30.0 g L^−1^), glucose (20.0 g L^−1^), KH_2_PO_4_ (1.0 g L^−1^), MgSO_4_ (1.0 g L^−1^), agar (10.0 g L^−1^) at 4°C. In a previous study, the isolated fungus growing on potato dextrose agar (PDA) medium (dextrose 20.0 g L^−1^, agar 15.0 g L^−1^, potato infusion 200.0 g L^−1^) was identified as *P. umbellatus* by means of molecular biological analysis of the internal transcribed region (ITS) [43]. Subsequently, *P. umbellatus* cultures were transferred from wheat bran agar slants to a wheat bran substrate.

### Reagent

Glucose, KH_2_PO_4_, MgSO_4_·7 H_2_O, agar, H_2_SO_4_, ascorbic acid and phenol were purchased from Kebio Biotechnology Co., Ltd of Beijing, China. Reagents used to detect intracellular ROS in the fungi, including 5-(and- 6)-chloromethyl-2',7'-dichlorodihydrofluorescein diacetate, acetyl ester (CM-H_2_DCFDA) and the diluent from the GMS10010.7 kit, were purchased from Genmed, Scientifics Inc., USA. Diphenyleneiodonium (DPI) and apocynin (Apo) were the products of Sigma.

Anhydrous D-Glucose, which was used as a chemical reference substance for the detection of polysaccharide content, was purchased from the Chinese National Institutes for Food and Drug Control.

### Substrate for the Sawdust-based Cultivation of *P. umbellatus*

The cultural medium components of corn (166.0 g L^−1^), sawdust (668.0 g L^−1^), wheat bran (166.0 g L^−1^), KH_2_PO_4_ (2.0 g L^−1^), MgSO_4_·7 H_2_O (0.1 g L^−1^) and sucrose (10.0 g L^−1^) were added to 1000 ml of deionized water and mixed evenly. The substrate was then divided into 100 grams aliquots in cyclindrical glass bottles with a diameter of 6.8 cm and autoclaved at 122°C for 120 min.

### *P. umbellatus* Cultivation

Three grams of *P. umbellatus* in wheat bran substrate was transferred into each of the glass bottles containing the sawdust-based substrate described above. The samples were then cultured at 25°C for 45 days in the dark. The samples were next divided randomly into low temperature group and room temperature groups. First, we screened the optimal conditions for *P. umbellatus* sclerotial formation in the low-temperature group. *P. umbellatus* was respectively cultured at different temperatures of −20°C and at one degree increments from 0°C to 20°C and for different periods of time at one day increments from 1 to 30 days. The fungal sclerotial formation was observed. After screening, we observed that cultivation at 5°C for 15 days’ cultivation was optimal for *P. umbellatus* sclerotial formation. Thus, for the low-temperature group, *P. umbellatus* was cultured at 5°C for 15 days and returned to 25°C for the subsequent experiments; in the room temperature group, *P. umbellatus* was cultured at 25°C for the duration of the experiment, with 30 replicates in each group. Morphogenesis and different macroscopically characteristics of sclerotial differentiation in *P. umbellatus* during cultivation were observed throughout the cultivation period. Each experiment was repeated three times.

### Determination of Polysaccharide Content of Artificial *P. umbellatus* Mycelia and Sclerotia and Wild Sclerotia

A previously described and modified phenol-sulfuric acid method [44] was used to determine polysaccharide content. This procedure was first conducted by Dubois et al in 1956 [45]. Prior to measuring the total carbohydrate content, a standard curve using anhydrous D-glucose as a chemical reference substance was generated as described previously [46]. Briefly, polysaccharides from *P. umbellatus* mycelia or sclerotia were extracted by degreasing and then purified by means of water extracting and ethanol precipitation. Samples containing certain polysaccharide in 1 ml water were mixed with 1 ml of 6% phenol. Five milliliters of concentrated sulfuric acid was added rapidly and evenly to each sample to generate the heat needed to drive the reaction. The reaction mixtures were then allowed to cool to room temperature (20°C), and the absorbance at 490 nm was measured in a spectrophotometer (Shanghai, model 580), with 10 replicates in each group. Deioned water was utilized as a blank. The experiment was repeated three times.

### Fluorescence Microscopy and Fluorescence Intensity of ROS in *P. umbellatus* Mycelial Cells

ROS detection was performed using a GMS10010.7 kit with a modified protocol. In the control group, surface samples were respectively taken from *P. umbellatus* mycelia cultured for 45, 60, 75, 90 and 105 days in the dark at 25°C using scotch tape. In the low-temperature group, surface samples of *P. umbellatus* mycelia or sclerotia were taken with scotch tape at the following time points: after cultivation for 45 days at 25°C, 15 days after cultivation at 5°C (total cultivation time 60 days), and 15 days after being transferred to 25°C again (total culture time 75 days). Subsequently, samples were performed at 15 days intervals until the total cultivation time was 105 days. The fluorescence staining was prepared by quickly mixing CM-H_2_DCFDA with the dilution from the GMS10010.7 kit at the volume ratio of 1∶20. Mixing was performed in the dark and on ice to avoid quenching of the CM-H_2_DCFDA fluorescent dye. Next, 20 µl of the prepared staining fluid was dropped onto individual glass slide in the dark. The samples adhered to scotch tape were placed face down on the slides so that they were in full contact with the staining fluid. The slides were then incubated at 28°C for 60 minutes in the biochemical incubator in the dark. Finally, labeled ROS in the mycelial cells from each sample were visualized using an AxioCan HRc Zeiss microscopy observed with the excitation wavelength of 490nm and an emission wavelength of 530nm. The fluorescence intensity was calculated as described previously [47]. Briefly, the intensities were quantified as gray values from 50 samples of three independent experiments during each cultivation stage using Axiol Vision Rel 4.6.

### Effect of Antioxidants on Sclerotial Differentiation

Before inoculation, exogenous sterile DPI (1, 2 and 3 mM) [35], Apo (10, 20 and 30 mM) and different concentrations of vitamin C (1–15 mg mL^−1^) were added to the growth medium in the final concentrations indicated. These groups were cultivated in the same manner as the low-temperature group for sclerotial formation. Sclerotial formation was observed during the cultivation period after the antioxidants were added. The experimental groups that received treatment were referred to as antioxidant complemented groups. No antioxidants were administrated to the low-temperature group, which was used as the control group in these experiments. Both the antioxidant complement groups and the control group were treated as previously described (25°C for 15 days, followed by 5°C for 15 days and return to 25°C for the continuous cultivation). The experiment was repeated three times, with 30 replicates in each group.

### Analysis of ROS Production in Mycelia after Treatment with Antioxidants

Fluorescence microscopy was performed for each group at 90 days after cultivation, and the fluorescence intensity of ROS in *P. umbellatus* mycelial cells was calculated.

### Microscopic Observation

*P. umbellatus* mycelia at different stages of cultivation were soaked in 10% KOH solution and subjected to morphological examination using Zeiss Axio Imager A1 microscope [48].

### Scanning Electron Microscopic Examination of the Artificial Sclerotia

The samples were prepared according to the protocol of Xing and Guo [44]. The sclerotia were cultivated for 120 days and fixed with 2.5% glutaraldehyde for 48 hours at 4°C. Subsequently, the samples were air-dried and sputtered-coated with gold palladium and observed and photographed using a JEOL JSM-6510 Scanning Electron Microscope.

### Microscopic Structure of *P. umbellatus* Sclerotia Using Paraffin Sectioning

Paraffin sectioning was performed as previously described [49]. Briefly, the artificial sclerotia formed at 105, 120, 150 days were fixed in FAA solution (formalin: glacial acetic acid: 50% ethanol at 1∶1∶18), dehydrated though a series of graded ethanol, embedded in paraffin and then sectioned at 10 µm with a rotary microtome.

### Data Analysis

The data were analyzed with T-test or one-way ANOVA and all statistical analyses were performed using SPSS 11.0 (SPSS, Chicago, IL, USA). Data were presented as means ± SD from at least three independent experiments. *P* values <0.05 were considered significant difference, *P*<0.01 was indicative of very significant difference.
